# Cpf1 enables fast and efficient genome editing in Aspergilli

**DOI:** 10.1186/s40694-019-0069-6

**Published:** 2019-05-01

**Authors:** Katherina Garcia Vanegas, Zofia Dorota Jarczynska, Tomas Strucko, Uffe Hasbro Mortensen

**Affiliations:** Eukaryotic Molecular Cell Biology, Section for Synthetic Biology, Department of Biotechnology and Biomedicine, Technical University of Denmark, Søltofts Plads, Kongens Lyngby, Denmark

**Keywords:** CRISPR, Cpf1, Gene targeting, Gene editing, Filamentous fungi, *Aspergillus*

## Abstract

**Background:**

CRISPR technology has revolutionized fungal genetic engineering by increasing the speed and complexity of the experiments that can be performed. Moreover, the efficiency of the system often allows genetic engineering to be introduced in non-model species. The efficiency of CRISPR gene editing is due to the formation of specific DNA double-strand breaks made by RNA guided nucleases. In filamentous fungi, only Cas9 has so far been used as the CRISPR nuclease. Since, gene editing with Cas9 is limited by its 5′-NGG-3′ protospacer adjacent motif (PAM) sequence, it is important to introduce RNA guided nucleases that depend on other PAM sequences in order to be able to target a larger repertoire of genomic sites. Cpf1 from *Lachnospiraceae bacterium* employs a PAM sequence composed of 5′-TTTN-3′ and therefore serves as an attractive option towards this goal.

**Results:**

In this study we showed that *Lb_cpf1* codon optimized for *Aspergillus nidulans* can be used for CRISPR based gene editing in filamentous fungi. We have developed a vector-based setup for Cpf1-mediated CRISPR experiments and showed that it works efficiently at different loci in *A. nidulans* and in *A. niger*. Specifically, we used our setup to demonstrate that Cpf1 is able to catalyze oligonucleotide-mediated genomic site-directed mutagenesis and marker-free gene targeting.

**Conclusions:**

In this paper we introduce Cpf1 as a new tool in the fungal CRISPR toolbox. Our experiments demonstrate that Cpf1 can be efficiently used in Aspergilli for gene editing thereby expanding the range of genomic DNA sequences that can be targeted by CRISPR technologies.

**Electronic supplementary material:**

The online version of this article (10.1186/s40694-019-0069-6) contains supplementary material, which is available to authorized users.

## Background

The rapid accumulation of fully sequenced fungal species [1–4] in combination with the development of efficient CRISPR based genome editing technology in a broad range of fungi [5–11] is fundamentally changing strategies for investigating fungal biology [12, 13]. Hence, experiments based on reverse genetics are no longer limited to a few model fungi where molecular biology toolboxes have been implemented, but can be performed in basically any host of interest. Even model fungi, where efficient gene targeting has been implemented via e.g. elimination of the non-homologous end-joining DNA repair pathway [14–16], benefits from CRISPR technology. For example, it sets the stage for complex and more ambitious experimental endeavors by facilitating multiplexing and marker-free gene manipulations including oligonucleotide directed gene deletion and site-directed mutagenesis [5, 17].

**Fig. 1:**
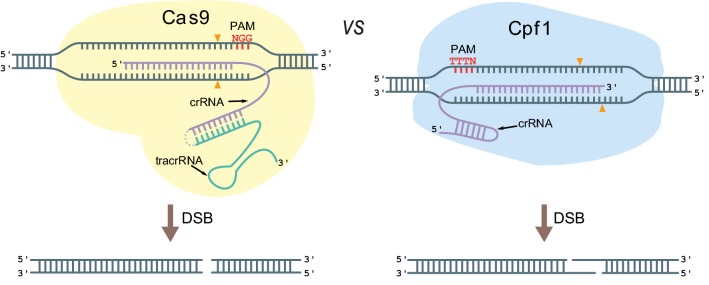
A comparison of CRISPR-Cas9 versus CRISPR-Cpf1 modes of action. Note that for Cas9, crRNA and tracrRNA are shown as a fused single-guide RNA. See text for details

The specificity of RNA guided CRISPR nucleases, see Fig. 1, is determined by the protospacer sequence in its guide RNA and protospacer adjacent motif, PAM, at the target site [18–20]. Hence, a restriction of CRISPR nucleases is the PAM sequence at the target site as the protospacer can be easily changed. This is particularly important for CRISPR techniques that depend on accurate positioning of the DNA double-strand break (DSB) for genetic engineering, e.g. for oligonucleotide directed mutagenesis or for tagging proteins with fluorescent proteins or purification tags via insertion of PCR fragments containing short targeting sequences. In case of the most commonly used CRISPR nuclease, the class 2 Sp_Cas9 from *Streptococcus pyogenes*, the limiting PAM sequence is a trinucleotide 5′-NGG-3′ (or less efficient 5′-NAG-3′) [18, 21]. To expand the range of possible target sites, it is therefore desirable to add other CRISPR nucleases with different PAM sequence requirements to the CRISPR toolbox.

Another class 2 CRISPR nuclease Cpf1 (also known as Cas12a [22]) from *Lachnospiraceae bacterium* has recently appeared as a promising addition to the CRISPR toolbox [23, 24]. This enzyme has several biochemical features that differ from those of Cas9, see Fig. 1. Here we highlight that the PAM sequence of Cpf1 is 5′-TTTN-3′ [23] and it therefore expands the repertoire of protospacers that can be used for CRISPR-mediated gene editing (Fig. 1). Moreover, Cpf1 creates five nucleotide 5′-OH staggered DNA DSB ends formed in the distal end of the target sequence relative to the PAM. Its protospacer is longer than that of Cas9 and it may therefore be more specific [23, 25]. As a genetic tool Cpf1 offers a simple system for gene editing as its crRNA, which contains the protospacer, directly functions as a guide RNA [23]. In contrast, with native Cas9, the crRNA needs to complex with tracrRNA to form the guide RNA [26]. In addition, some species may prefer to produce Cpf1 rather than Cas9 for reasons that may not be easy to predict. In this report, we present a vector-based Cpf1 platform for fungal CRISPR-mediated gene editing and demonstrate for the first time that Cpf1 can be efficiently used for gene editing in filamentous fungi.

## Results and discussion

### A flexible gene editing system based on Cpf1

**Fig. 2:**
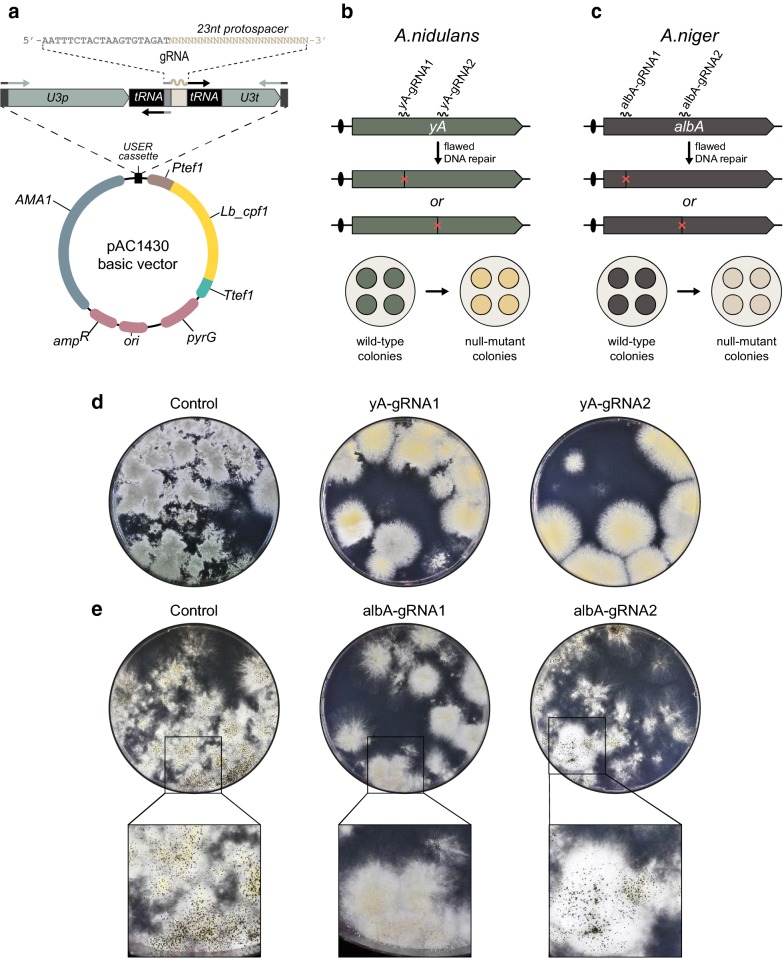
An efficient Cpf1 system for fungal gene editing. **a** Illustration of vector pAC1430 expressing Lb_Cpf1 and how to insert a gRNA expression cassette. **b**, **c** Schematic overview of the positions of Cpf1-gRNA target sites in *A. nidulans* (**b**) and in *A. niger* (**c**) used for assessing Cpf1 functionality. **d**, **e** Examples of transformation plates for assessment of Cpf1-gRNA functionality in Cpf1-mediated gene mutation experiments in *A. nidulans* (**d**) and in *A. niger* (**e**). pAC1430 was used in control experiments whereas pAC1430 derivatives, expressing specific gRNA genes as indicated above plates, were used to induce locus specific mutagenesis

To set the stage for simple Cpf1-mediated gene editing in *Aspergillus*, we first acquired a variant of *L. bacterium cpf1* codon optimized for expression in *A. nidulans* and extended with an SV40 nuclear localization sequence (NLS), see “Methods”. This gene was equipped with *A. nidulans tef1* promoter and terminator and inserted into an *Af_pyrG*-AMA1 based vector containing an empty USER cassette, to produce pAC1430, see Fig. 2a. gRNAs for Cpf1 were generated using a setup we have previously developed for Cas9-mediated gene editing [17]. Briefly, a gene cassette containing one or more gRNA sequences spaced by tRNA and under the control of polymerase III promoter *Af_U3p* and *Af_U3t* terminator can be assembled from PCR fragments. The PCR fragments are generated by using the plasmid pFC902 as template and inserted into the USER cassette of pAC1430 by USER fusion or another similar cloning method that allows seamless joining of DNA fragments (for details see Additional file 1: Fig. S1). The resulting Cpf1-CRISPR-tRNA vector is ready for transformation into a *pyrG*^−^ strain. Cpf1-CRISPR vectors containing *argB*, *hygB* and *ble* as markers were also made to allow transformation into other hosts, see “Methods”.

### Cpf1-gRNA nucleases efficiently cut specific fungal genes in vivo

To investigate whether our Cpf1 variant is feasible for flexible gene editing we used the *pyrG* based Cpf1-CRISPR-vector. First we tested whether it could target and cut different pigment marker genes in *A. nidulans* and in *A. niger*. Since not all gRNAs are expected to guide Cpf1 to a target gene, we constructed four Cpf1-CRISPR-tRNA vectors each expressing *cpf1* and one of two specific gRNA genes (*yA*-*gRNA1* or *yA*-*gRNA2*) matching the *yA* ORF in *A. nidulans;* and, one of two specific gRNA genes (*albA*-*gRNA1* or *albA*-*gRNA2*) matching the *albA* ORF in *A. niger* (Fig. 2b, c). If *yA* is cut and subsequently destroyed due to error-prone DNA repair, *A. nidulans* develops yellow colonies as nonfunctional yA laccase fails to polymerize a yellow polyketide, YWA1, in the conidia into a green pigment [27]. If *albA* is destroyed, *A. niger* develops white colonies as YWA1 is not formed (Fig. 2b, c) [28]. We also constructed a control Cpf1-CRISPR plasmid encoding no gRNA.

When the empty Cpf1-CRISPR plasmid was transformed into NHEJ proficient *A. nidulans* and *A. niger* strains, transformants were easily obtained. As expected these transformants produced green and black conidia, respectively, as no Cpf1-gRNA complexes were formed in these transformants (Fig. 2d, e). In contrast, when the Cpf1-CRISPR-tRNA plasmids expressing *yA*-*gRNA1* or *yA*-*gRNA2* were individually transformed into *A. nidulans*, mostly yellow colonies were obtained. This result indicates that the *yA* gene was efficiently cut by Cpf1 in a manner depending on yA-gRNA1 and yA-gRNA2 and subsequently destroyed due to flawed DNA repair (Fig. 2d). Similarly, with *A. niger*, white mutant colonies were easily obtained when Cpf1-CRISPR-tRNA plasmid expressing *albA*-*gRNA1* was used for transformation (Fig. 2e). With the Cpf1-CRISPR-tRNA plasmid expressing *albA*-*gRNA2*, no white transformants were achieved. The latter experiment indicates that albA-gRNA2, unlike albA-gRNA1, is an inefficient gRNA. Accordingly, for the next set of experiments we only used the Cpf1-CRISPR-tRNA plasmids expressing *yA*-*gRNA1*, *yA*-*gRNA2*, and *albA*-*gRNA1*.

### Cpf1 efficiently stimulates single-stranded oligonucleotide directed genomic site-specific mutagenesis

**Fig. 3:**
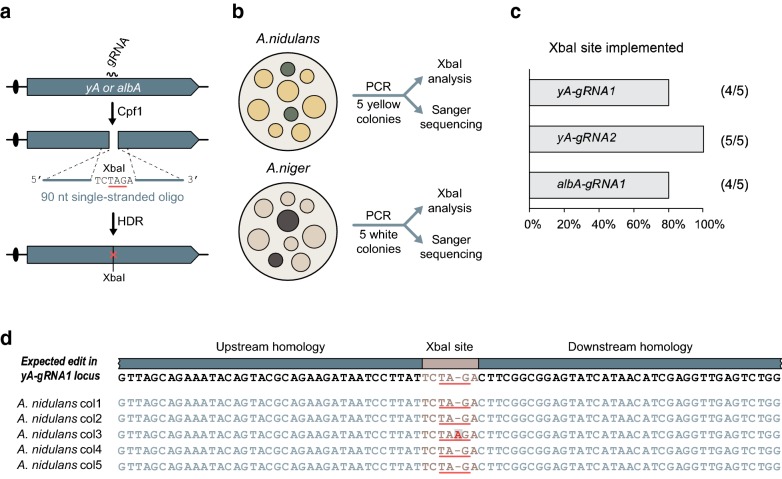
Cpf1-gRNA catalyzes single-stranded oligonucleotide-mediated site specific mutagenesis in the genome. **a** Schematic overview of the experimental setup to test for oligonucleotide-mediated mutagenesis. The position of the amber stop-codon is underlined in red. **b** Strategies to validate the Cpf1 mediate genetic edits in *A. nidulans* and *A. niger*. **c** Efficiency of gene editing at three different sites located in *yA* and *albA*. **d** Sequencing results of five yellow *A. nidulans* transformants generated by mutagenesis at the yA-gRNA1 target site. Unexpected nucleotide insertion in colony three is indicated in red. *HDR* homology directed recombination

We have previously observed that single-stranded oligonucleotides can be efficiently used as templates for the repair of Cas9 induced DNA DSBs in NHEJ deficient Aspergilli and used this capacity to introduce specific mutations into the genome of *A. nidulans* and *A. niger* [17]. We therefore tested whether DNA DSBs produced by Cpf1 can be repaired in the same manner. Accordingly, we co-transformed NHEJ deficient *A. nidulans* and *A. niger* strains with the Cpf1-CRISPR-tRNA plasmids presented above and relevant single-stranded repair oligonucleotides. Specifically, we used 90-base long oligonucleotides matching different Cpf1-gRNA cut sites in *yA* and in *albA* in a symmetric manner (see Fig. 3a). The repair oligonucleotides were all designed to introduce an amber stop codon and an XbaI site into the target gene at the position cleaved by Cpf1.

When the empty Cpf1-CRISPR plasmid was transformed into *A. nidulans* or *A. niger* in the presence of the relevant repair oligonucleotides, none of the transformants showed any conidia pigment phenotype (Additional file 2: Fig. S2). This result reflects that in the absence of Cpf1 induced DNA DSBs in *yA* or in *albA* these oligonucleotides do not introduce genetic alterations in these genes. In contrast, when Cpf1-CRISPR-tRNA vectors expressing either *yA*-*gRNA1* or *yA*-*gRNA2* were co-transformed with repair oligonucleotides matching the corresponding break sites in *yA*, all transformants formed yellow colonies (Additional file 2: Fig. S2). Similarly, when the Cpf1-CRISPR-tRNA vector expressing *albA*-*gRNA1* was co-transformed with a repair oligonucleotide matching the albA-gRNA1 target site, all transformants formed white colonies (Additional file 2: Fig. S2). We next tested whether the phenotypes were caused by DNA repair events involving the oligonucleotides as repair templates. Accordingly, from each of the three experiments, five randomly picked transformants displaying a color phenotype were streak purified; and for each of the resulting strains, the relevant sequence either at the *yA* or the *albA* locus was amplified by PCR and analyzed by XbaI digestion for the presence of the mutation (see Fig. 3b). XbaI digestions of the PCR fragments representing the three different genomic positions showed that the restriction site has been introduced into *A. nidulans yA* or into *A. niger albA* in 13 out of the 15 cases analyzed (Fig. 3c and Additional file 3: Fig. S3). More importantly, sequencing of PCR fragments from these 13 colonies demonstrated that they only contained the desired gene edits. In the remaining cases, one in *yA* and one in *albA*, the oligonucleotides were still used as repair templates, but the intended XbaI sites were accompanied by additional mutation. With the *yA* mutant, we observed insertion of an additional adenine nucleotide residue, while with the *albA* mutant, a guanine-residue was substituted by an adenine residue. In both cases, the additional mutation destroyed the XbaI site and introduced an ochre stop-codon explaining the conidia color phenotypes (see Fig. 3d and Additional file 4: Fig. S4). We conclude that Cpf1 can be efficiently used for genomic site-directed mutagenesis based on oligonucleotide-mediated repair in Aspergilli.

### Cpf1 efficiently stimulates selection-free gene targeting

**Fig. 4:**
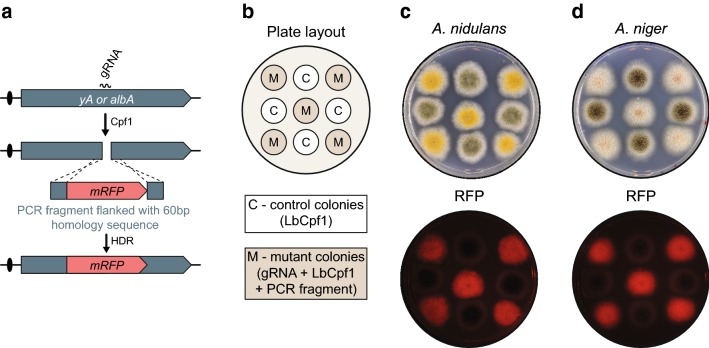
Cpf1-gRNA catalyzes gene disruption by gene targeting. **a** Schematic overview of the experimental setup to test for Cpf1-gRNA-mediated gene disruption. **b** Plate layout to validate correct gene disruption in *A. nidulans* and *A. niger*. **c**, **d** visual inspection of *A. nidulans* and *A. niger* colonies, respectively, for development of color and production of red fluorescence

To investigate whether Cpf1 supports selection-free insertion of a gene into a defined site in the genome, we used the same Cpf1-CRISPR-tRNA vectors to integrate *mRFP* into either *yA* or *albA*. Inspired by the fact that short oligonucleotides can be used for Cpf1-mediated mutagenesis, we tested whether *mRFP* PCR fragments containing 60 base-pair targeting sequences could be integrated into *yA* or *albA* (Fig. 4a and “Methods”).

Co-transformation of NHEJ deficient *A. nidulans or A. niger* strains with Cpf1-CRISPR-tRNA vectors expressing either *yA*-*gRNA2* or *albA*-*gRNA1* along with the proper *RFP* gene-targeting PCR fragment produced only yellow or white colonies, respectively (see Additional file 5: Fig. S5). The mutant color phenotypes were dependent on formation of Cpf1 induced DNA DSBs in *yA* and *albA* as co-transformation of the two strains with the *RFP* gene-targeting PCR fragments and an empty Cpf1-CRISPR plasmid only produced transformants with wild-type pigments.

To investigate whether the yellow and white phenotypes were caused by the desired insertion of *mRFP* into *yA* and *albA*, respectively, we randomly picked five colonies from each experiment for fluorescence imaging (Fig. 4b). Direct inspection of the plates by a fluorescence detection system (see Additional file 6: Fig. S6 and “Methods”) demonstrated that all color mutants produced RFP signals, whereas control colonies with wild-type colors did not fluoresce (Fig. 4c, d). For the same color mutants, we next validated by diagnostic PCR that *yA* and *albA* were indeed disrupted in *A. nidulans* and in *A. niger* as the result of the predicted gene-targeting events (Additional file 7: Fig. S7). Together these results strongly indicate that Cpf1 induced DNA DSBs can be used efficiently to stimulate marker-free gene targeting in Aspergilli.

### Cpf1-mediated gene integration into defined expression sites

We have previously identified and characterized defined expression sites in *A. nidulans* (*Anid_IS1*) and *A. niger* (*Anig_IS1*), which are located in intergenic regions of their genomes [29, 30]. Targeting into these sites does not produce any visual phenotype to facilitate detection of gene integration events. Since it was possible to efficiently integrate PCR based *RFP* fragments into visual marker genes in *A. nidulans* and *A. niger* via Cpf1-gRNA-mediated breaks, we envisioned that Cpf1-gRNA could also mediate targeted gene integration into these expression sites in a marker-free manner to facilitate use of the sites for heterologous gene expression. Considering that no visible phenotype would accompany gene targeting into these loci, we first identified efficient gRNAs for the two integrations sites. We have previously observed that efficient sgRNAs for Cas9 can be identified in an experimental setup we call TAPE (Technique to Assess Protospacer Efficiency) [17, 31]. TAPE is based on the simple principle that the combination of Cas9 and efficient sgRNAs produce DNA DSBs that are lethal if they remain unrepaired. Indeed, in the absence of a homologous repair template, we have observed that transformation of fungi with CRISPR vectors encoding Cas9 and efficient sgRNAs produce numbers of transformants that are significantly lower as compared to the numbers obtained with empty CRISPR vectors that do not encode any sgRNAs [17, 31]. Importantly, this effect is enhanced if the experiment is performed in an NHEJ deficient strain and such strains are therefore preferred for TAPE experiments [17, 31].

**Fig. 5:**
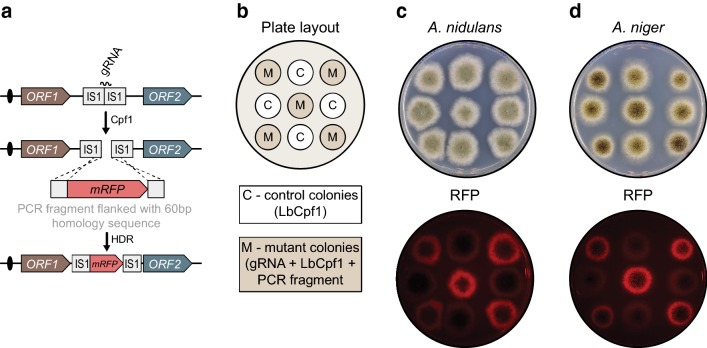
Cpf1-gRNA-mediated gene integration into a specific gene expression site. **a** Schematic overview of the experimental setup for marker-free specific Cpf1-gRNA-mediated gene insertion. **b** Plate layout to validate correct gene insertion in *A. nidulans* and *A. niger*. **c**, **d** visual inspection of *A. nidulans* and *A. niger* colonies, respectively, for development of color and production of red fluorescence. *IS1* (Integration site 1, see text)

To investigate whether TAPE also applies to RNA guided Cpf1 nucleases, we constructed a set of Cpf1-CRISPR-tRNA vectors expressing five different gRNAs matching *Anid_IS1* and four matching *Anig_IS1*, Additional file 8: Fig. S8. All relevant Cpf1-CRISPR-tRNA vectors were transformed into NHEJ proficient and deficient *A. nidulans* and *A. niger* strains and the numbers of transformants were compared. These analyses suggests that with *A. nidulans* strains, all five gRNAs are efficient whereas with *A. niger* strains, two (Anig_IS1-gRNA2 and Anig_IS1-gRNA3) out of four are efficient. We chose Anid_IS1-gRNA4 and Anig_IS1-gRNA2 for further experimentation and co-transformed NHEJ deficient strains of *A. nidulans* and *A. niger* with the relevant Cpf1-CRISPR-tRNA vectors and PCR generated linear gene-targeting substrates containing *RFP* flanked by 60 base pairs of up- and down-stream sequences matching either *Anid_IS1* or *Anig_IS1* (see Fig. 5a). Next, for each species, five transformants were randomly selected and restabbed onto solid medium before further analysis (see Fig. 5b). In agreement with efficient Cpf1-mediated integration of *RFP* into *Anid_IS1* and *Anig_IS1*, all the restabbed transformants contained a robust RFP signal (see Fig. 5c, d). In contrast, the control colonies produced from co-transformation of the strains with the RFP fragments and empty Cpf1-CRISPR vector did not show any red fluorescence. Diagnostic PCR reactions of the ten restabbed mutant colonies confirmed that *RFP* has been integrated into *Anid_IS1* and *Anig_IS1* in all cases (Additional file 7: Fig. S7). Cpf1 can therefore be used to mediate efficient marker-free gene integration of PCR fragments into phenotypically neutral integration sites.

## Conclusions

We have shown that Cpf1 can be efficiently used in fungi for marker-free gene editing using repair templates based on single-stranded oligonucleotides or PCR fragments containing very short targeting sequences. Since Cpf1 uses a fundamentally different PAM sequence than the one used by Cas9, the chance of finding a functional protospacer in the genome at a desirable site has been greatly increased. This will particular assist in processes where the position of the DNA break is important, e.g. for oligonucleotide directed mutagenesis or for introduction of gene extensions for protein tagging. Moreover, when introducing CRISPR into a non-model fungus we envision that some fungi may prefer Cpf1 over Cas9 (or vice versa) for DNA DSB formation. A larger repertoire of CRISPR nucleases may therefore speed up transferring CRISPR technology to non-model fungi. In this report we only tested single genome edits with Cpf1, but our methods are extendable for multiplexing since the gRNA cloning system is compatible with the system we have previously used for multiplexing with Cas9-CRISPR vectors [17]. Cpf1 has been reported to display very low off target effects in human cells [25, 32] and plants [33], but since the chances of inducing off target effects will be unique for each protospacer, it is recommendable to be cautious and either sequence the final strains or analyze several transformants to substantiate conclusions on a phenotype. In conclusion we envision that Cpf1 could serve as a main CRISPR nuclease for fungal gene editing.

## Methods

### Strains and media

All plasmids were propagated in *Escherichia coli* (*E. coli*) strain DH5α. After transformation DH5α cells were cultured for 12 h at 37 °C on Luria broth (LB) plates, prepared with 25 g/l of LB with agar and supplemented with 100 μg/ml ampicillin. Plasmid rescue cultivations were prepared using liquid LB media prepared with 25 g/l LB Broth and supplemented with 100 μg/ml ampicillin.

The main Aspergilli strains used in this study are summarized in Table 1. All Aspergilli were cultivated on standard solid glucose based minimal medium (MM) (1% glucose, 1 × nitrate salt solution, 0.001% Thiamine, 1x trace metal solution, 2% agar), supplemented when required with 10 mM uridine (Uri), 10 mM uracil (Ura), and/or 4 mM l-arginine (Arg). Transformation media (TM) was prepared as MM media, apart from glucose, which was replaced with 1 M sucrose.

**Table 1:** Fungal strains used in this study

Species	Strain name	IBT^a^ number	Genotype	References
*A. nidulans*	NID1	29539	*argB2*, *pyrG89*, *veA1*, *nkuAΔ*	[15]
*A. nidulans*	NID5	27263	*argB2*, *pyrG89*, *veA1*	
*A. niger*	NIG81		*pyrG1*	[17]
*A. niger*	NIG96		*pyrG1*, *kusAΔ*	[17]

^a^IBT culture collection of fungi

### PCR and assembly of plasmids by USER cloning

Primers for PCR were obtained from Integrated DNA Technologies (IDT) and their sequences can be found in Additional file 9: Table S1. All PCR products for cloning purposes were amplified in 35 cycles using proof-reading Phusion polymerase (Thermo Fisher Scientific) following the instructions of the supplier. Standard reaction volumes were 50 μl including 25 μl Phusion U Hot Start PCR Master Mix, 0.5 μM primers, 10–50 ng plasmid template and MilliQ water to reach the desired final volume. Diagnostic PCR reactions were performed using DNA from conidia as template and MyTaq™ Plant-PCR Kit (Bioline). Prior to PCR reaction, spore suspensions were prepared by adding spores into 20 μl MilliQ water. The resulting samples were then microwaved at 800 Watts for 3 min. Standard reaction volumes for diagnostic PCRs were 20 μl including 0.5 μM primers, 10 μl MyTaq™ Plant-PCR Kit, 3 μl of spore suspension and MilliQ water to reach the final volume.

All vectors were assembled by USER cloning [34] and can be found in Additional file 10: Table S2. The *Lb_cpf1* gene encoding *L. bacterium* Cpf1 was codon optimized for translation in *A. nidulans* and synthetized by GeneArt™ (Thermo Fisher Scientific) (see Additional file 11: Fig. S9). An USER compatible backbone encoding the *Lb_cpf1* gene was created by fusing it with *A. nidulans tef1* promoter and terminator and then incorporating into pAC572 USER digested backbone. The primer fusing the *tef1* promoter with the digested backbone included a new USER restriction site (PacI/Nt.BbvCI USER cassette). The newly created Cpf1-CRISPR USER compatible backbone (pAC1430) was in subsequent experiments used to create vectors encoding the gRNA expression cassette. The gRNA cassette was constructed using two PCR products with USER sticky ends, the U3p-tRNA-repeat and the protospacer-tRNA-U3t. The PCR products were amplified using vector pFC902 [17]. Three additional Cpf1-CRISPR vectors with different selective markers: pAC1748 (*argB*), pAC1749 (*hygB*), and pAC1750 (*ble*) were constructed by inserting a PCR fragment containing the *Ptef1*::*Lb_cpf1::Ttef1* into USER cassette of pAC573, pAC574 and pAC575, respectively. The *Lb_cpf1* PCR fragment was generated by primes P174 and P58 using pAC1430 as a template. pAC572, pAC573, pAC574, pAC575, pAC1430, pAC1748, pAC1749 and pAC1750 are available from Addgene collection.

### Transformation and strain validation by diagnostic PCR

Protoplasts were generated as described by Nielsen et al. [35]. For transformation approximately 10^7^ protoplasts and 0.5 μg of Cpf1-CRISPR-tRNA vector were mixed with 150 μl PCT solution and incubated for 10 min at room temperature, followed by adding of 250 μl ATB and plating on 1 M sucrose based TM with selection. All TM plates were incubated at 30 °C for *A. niger* and 37 °C for *A. nidulans*. In gene-editing experiments we either added as a repair template, 1 μg of oligonucleotides (IDT, see Additional file 12: Table S3) or 1 μg of RFP gene-targeting PCR fragment, together with 0.5 μg of Cpf1-CRISPR-tRNA vector.

Target specific genome engineering was analyzed by diagnostic PCR. Primers for detecting precise gene editing mutations were designed to bind up- and downstream from the gene-targeting sequence or within the PCR construct. The amplified bands for gene-targeting experiments with oligonucleotides were subsequently purified with illustra GFX PCR DNA and Gel Band Purification Kit (GE Healthcare Bio-Sciences) and sent for sequencing (StarSEQ).

### Fluorescence photography

Red fluorescence was examined using an in-house build digital camera setup. Images of agar plates with either *A. nidulans* or *A. niger* colonies were captured using an SLR camera (Nikon D90) equipped with Light and Filter set (NIGHTSEA™), for details see Additional file 6: Fig. S6. Camera settings for fluorescent images were as follows: ISO Speed—ISO250, F-stop—f/7.1, Exposure time—1.6 s, Focal length—60 mm. Images were saved in JPEG format with a 300 dpi resolution.

## Additional files

**Additional file 1: Fig. S1.** Cloning procedure of tRNA based gRNA expression. **a** Primer pair sets for amplifying the bipartite gRNA biobricks. **b** Bipartite gRNA biobricks after PCR amplification. We note that one biobrick (P1 + 2) is constant for all experiments, as only the PCR fragment (Px + 4) containing the protospacer needs to be specifically produced for each new experiment. **c** Design of the primer tails for USER fusion of bipartite gRNA biobricks. **d** Cpf1-CRISPR vector fragment (pAC1430) after linearization with PacI and Nt.BbvCI. **e** Insertion of gRNA biobrick into Cpf1-CRISPR vector (pAC1430) by USER cloning in *E. coli*.

**Additional file 2: Fig. S2.** Repair of Cpf1 induced DNA DSBs in yA and albA using oligonucleotides as repair templates. Co-transformations of NHEJ deficient *A. nidulans* and *A. niger* strains. Panels above, co-transformations with Cpf1-CRISPR-tRNA vectors with gRNAs as indicated; panels below, co-transformations with empty Cpf1-CRISPR vectors. Repair templates are indicated below plates.

**Additional file 3: Fig. S3.** Validation of oligonucleotide-mediated mutagenesis in *A. nidulans* yA and *A. niger* albA by restriction enzyme digest. **a** PCR fragments covering the mutagenized region were produced transformants with a color phenotype. The sizes of the three individual PCR fragments generated for each of the three positions mutagenized are indicated below blue boxes. Successful implementation of the mutation creates an XbaI site and individual blue boxes indicate fragment sizes after XbaI digest of fragments that contain the mutation. **b** Agarose gel electrophoresis analysis of PCR fragments after XbaI digestion. Samples obtain from yA (two sites; positions are indicated by gRNA name) and albA transformants are indicated.

**Additional file 4: Fig. S4.** Sanger sequencing validation of two different loci mutated by oligonucleotide-mediated repair of Cpf1 induced DNA DSBs in *A. nidulans* yA (two different sites) and *A. niger* albA. Position of the repair oligonucleotide is indicated above sequencing results. Unexpected mutations are highlighted with the red box.

**Additional file 5: Fig. S5.** Figure S5 Repair of Cpf1 induced DNA DSBs in yA and albA using PCR fragments as repair fragments. Co-transformations of NHEJ deficient *A. nidulans* and *A. niger* strains. Panels above, co-transformations with Cpf1-CRISPR-tRNA vectors with gRNAs as indicated; panels below, co-transformations with empty Cpf1-CRISPR vectors. Repair templates, PCR fragments containing mRFP and flanked by 60 bp of targeting sequences specific for *A. nidulans* yA and *A. niger* albA, are indicated below plates.

**Additional file 6: Fig. S6.** Camera setup for qualitative plate screening by fluorescence photography. **a** The components of the setup; 1—Nikon D90 SLR camera, 2—Nikon AF-S Micro NIKKOR 60 mm lens, 3—lens attachment equipped with red filter (Nightsea™), 4—green light source (Nightsea™), 5—black glass background. **b** The camera setup in operational mode.

**Additional file 7: Fig. S7.** Diagnostic PCR validation of mRFP gene insertions into different genetic loci in *A. nidulans* and in *A. niger*. **a** Schematic drawing of the experimental setup. Small arrows indicate primers positions and Roman numbers indicate resulting PCR fragments. The expected fragment lengths for four different loci are summarized in the table. **b** Diagnostic PCR reactions analyzed by gel electrophoresis. Samples from all individual experiments are loaded as indicated above individual lanes.

**Additional file 8: Fig. S8.** TAPE experiment. Tests to assess protospacer efficiency of gRNAs targeting *A. nidulans* Anid_IS1 and *A. niger* Anig_IS1. Transformation of *A. nidulans* NHEJ proficient NID5 and NHEJ deficient NID1, as well as of *A. niger* NHEJ proficient NIG81 and NHEJ deficient NIG96 with empty Cpf1-CRISPR vector (pAC1430) or Cpf1-CRISPR-tRNA vectors encoding gRNAs as indicated. Plasmids used for each transformation are indicated below plates for NHEJ proficient strains and above plates for NHEJ deficient strains.

**Additional file 9: Table S1.** Primer used in this study.

**Additional file 10: Table S2.** Plasmids used in this study.

**Additional file 11: Fig. S9.** Sequence of *A. nidulans* codon optimized *Lb*_*cpf1*.

**Additional file 12: Table S3.** Oligonucleotides used in this study.

## Abbreviations

CRISPR: clustered regularly interspaced short palindromic repeats
crRNA: CRISPR RNA
DSB: double-strand brake
gRNA: guide RNA
NLS: nuclear localization sequence
PAM: protospacer adjacent motif
sgRNA: single guide RNA
TAPE: technique to assess protospacer efficiency
tracrRNA: trans-activating CRISPR RNA
