# Supplementary material for: Cpf1 enables fast and efficient genome editing in Aspergilli
Source: Fungal Biol Biotechnol. 2019 May 1;6:6. doi: 10.1186/s40694-019-0069-6 (PMC6492335; doi:10.1186/s40694-019-0069-6)
Supplement: Supplementary file 1 — Additional file 1: Fig. S1. Cloning procedure of tRNA based gRNA expression. a Primer pair sets for amplifying the bipartite gRNA biobricks. b Bipartite gRNA biobricks after PCR amplification. We note that one biobrick (P1 + 2) is constant for all experiments, as only the PCR fragment (Px + 4) containing the protospacer needs to be specifically produced for each new experiment. c Design of the primer tails for USER fusion of bipartite gRNA biobricks. d Cpf1-CRISPR vector fragment (pAC1430) after linearization with PacI and Nt.BbvCI. e Insertion of gRNA biobrick into Cpf1-CRISPR vector (pAC1430) by USER cloning in E. coli. [file 40694_2019_69_MOESM1_ESM.docx]

**Figure** **S1** Cloning procedure of tRNA based gRNA expression. A) Primer pair sets for amplifying the bipartite gRNA biobricks. B) Bipartite gRNA biobricks after PCR amplification. We note that one biobrick (P1+2) is constant for all experiments, as only the PCR fragment (Px+4) containing the protospacer needs to be specifically produced for each new experiment. C) Design of the primer tails for USER fusion of bipartite gRNA biobricks. D) Cpf1-CRISPR vector fragment (pAC1430) after linearization with PacI and Nt.BbvCI. E) Insertion of gRNA biobrick into Cpf1-CRISPR vector (pAC1430) by USER cloning in *E. coli*.
