# Supplementary material for: Cpf1 enables fast and efficient genome editing in Aspergilli
Source: Fungal Biol Biotechnol. 2019 May 1;6:6. doi: 10.1186/s40694-019-0069-6 (PMC6492335; doi:10.1186/s40694-019-0069-6)
Supplement: Supplementary file 2 — Additional file 2: Fig. S2. Repair of Cpf1 induced DNA DSBs in yA and albA using oligonucleotides as repair templates. Co-transformations of NHEJ deficient A. nidulans and A. niger strains. Panels above, co-transformations with Cpf1-CRISPR-tRNA vectors with gRNAs as indicated; panels below, co-transformations with empty Cpf1-CRISPR vectors. Repair templates are indicated below plates. [file 40694_2019_69_MOESM2_ESM.docx]

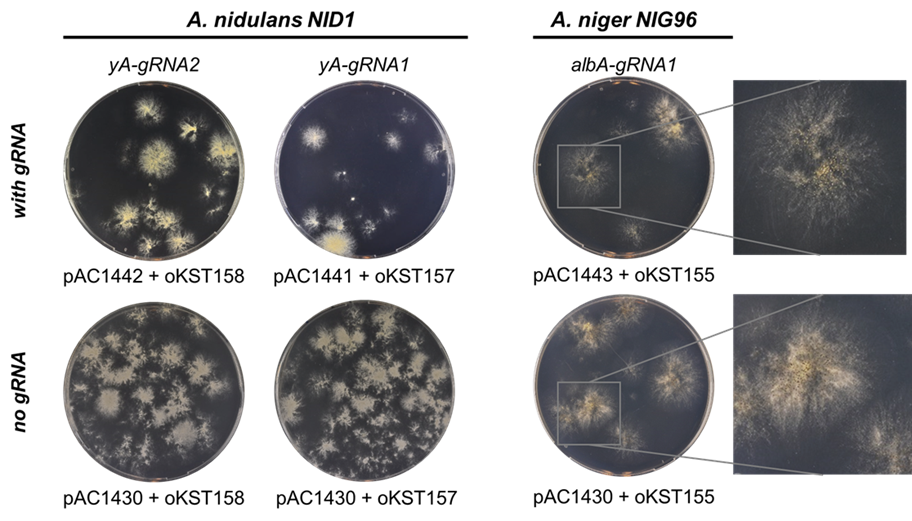


**Figure S2** Repair of Cpf1 induced DNA DSBs in *yA* and *albA* using oligonucleotides as repair templates. Co-transformations of NHEJ deficient *A. nidulans* and *A. niger* strains. Panels above, co-transformations with Cpf1-CRISPR-tRNA vectors with gRNAs as indicated; panels below, co-transformations with empty Cpf1-CRISPR vectors. Repair templates are indicated below plates.
