# Supplementary material for: Cpf1 enables fast and efficient genome editing in Aspergilli
Source: Fungal Biol Biotechnol. 2019 May 1;6:6. doi: 10.1186/s40694-019-0069-6 (PMC6492335; doi:10.1186/s40694-019-0069-6)
Supplement: Supplementary file 3 — Additional file 3: Fig. S3. Validation of oligonucleotide-mediated mutagenesis in A. nidulans yA and A. niger albA by restriction enzyme digest. a PCR fragments covering the mutagenized region were produced transformants with a color phenotype. The sizes of the three individual PCR fragments generated for each of the three positions mutagenized are indicated below blue boxes. Successful implementation of the mutation creates an XbaI site and individual blue boxes indicate fragment sizes after XbaI digest of fragments that contain the mutation. b Agarose gel electrophoresis analysis of PCR fragments after XbaI digestion. Samples obtain from yA (two sites; positions are indicated by gRNA name) and albA transformants are indicated. [file 40694_2019_69_MOESM3_ESM.docx]

**Figure S3** Validation of oligonucleotide-mediated mutagenesis in *A. nidulans yA* and *A. niger albA* by restriction enzyme digest. A) PCR fragments covering the mutagenized region were produced transformants with a color phenotype. The sizes of the three individual PCR fragments generated for each of the three positions mutagenized are indicated below blue boxes. Successful implementation of the mutation creates an XbaI site and individual blue boxes indicate fragment sizes after XbaI digest of fragments that contain the mutation. B) Agarose gel electrophoresis analysis of PCR fragments after XbaI digestion. Samples obtain from *yA* (two sites; positions are indicated by gRNA name) and *albA* transformants are indicated.
