# Supplementary material for: Cpf1 enables fast and efficient genome editing in Aspergilli
Source: Fungal Biol Biotechnol. 2019 May 1;6:6. doi: 10.1186/s40694-019-0069-6 (PMC6492335; doi:10.1186/s40694-019-0069-6)
Supplement: Supplementary file 4 — Additional file 4: Fig. S4. Sanger sequencing validation of two different loci mutated by oligonucleotide-mediated repair of Cpf1 induced DNA DSBs in A. nidulans yA (two different sites) and A. niger albA. Position of the repair oligonucleotide is indicated above sequencing results. Unexpected mutations are highlighted with the red box. [file 40694_2019_69_MOESM4_ESM.docx]

**Figure S4** Sanger sequencing validation of two different loci mutated by oligonucleotide-mediated repair of Cpf1 induced DNA DSBs in *A. nidulans yA* (two different sites) and *A. niger albA*. Position of the repair oligonucleotide is indicated above sequencing results. Unexpected mutations are highlighted with the red box.
