# Supplementary material for: Cpf1 enables fast and efficient genome editing in Aspergilli
Source: Fungal Biol Biotechnol. 2019 May 1;6:6. doi: 10.1186/s40694-019-0069-6 (PMC6492335; doi:10.1186/s40694-019-0069-6)
Supplement: Supplementary file 5 — Additional file 5: Fig. S5. Figure S5 Repair of Cpf1 induced DNA DSBs in yA and albA using PCR fragments as repair fragments. Co-transformations of NHEJ deficient A. nidulans and A. niger strains. Panels above, co-transformations with Cpf1-CRISPR-tRNA vectors with gRNAs as indicated; panels below, co-transformations with empty Cpf1-CRISPR vectors. Repair templates, PCR fragments containing mRFP and flanked by 60 bp of targeting sequences specific for A. nidulans yA and A. niger albA, are indicated below plates. [file 40694_2019_69_MOESM5_ESM.docx]

**Figure S5** Repair of Cpf1 induced DNA DSBs in *yA* and *albA* using PCR fragments as repair fragments. Co-transformations of NHEJ deficient *A. nidulans* and *A. niger* strains. Panels above, co-transformations with Cpf1-CRISPR-tRNA vectors with gRNAs as indicated; panels below, co-transformations with empty Cpf1-CRISPR vectors. Repair templates, PCR fragments containing *mRFP* and flanked by 60 bp of targeting sequences specific for *A. nidulans* *yA* and *A. niger albA*, are indicated below plates.
