# Supplementary material for: Cpf1 enables fast and efficient genome editing in Aspergilli
Source: Fungal Biol Biotechnol. 2019 May 1;6:6. doi: 10.1186/s40694-019-0069-6 (PMC6492335; doi:10.1186/s40694-019-0069-6)
Supplement: Supplementary file 6 — Additional file 6: Fig. S6. Camera setup for qualitative plate screening by fluorescence photography. a The components of the setup; 1—Nikon D90 SLR camera, 2—Nikon AF-S Micro NIKKOR 60 mm lens, 3—lens attachment equipped with red filter (Nightsea™), 4—green light source (Nightsea™), 5—black glass background. b The camera setup in operational mode. [file 40694_2019_69_MOESM6_ESM.docx]

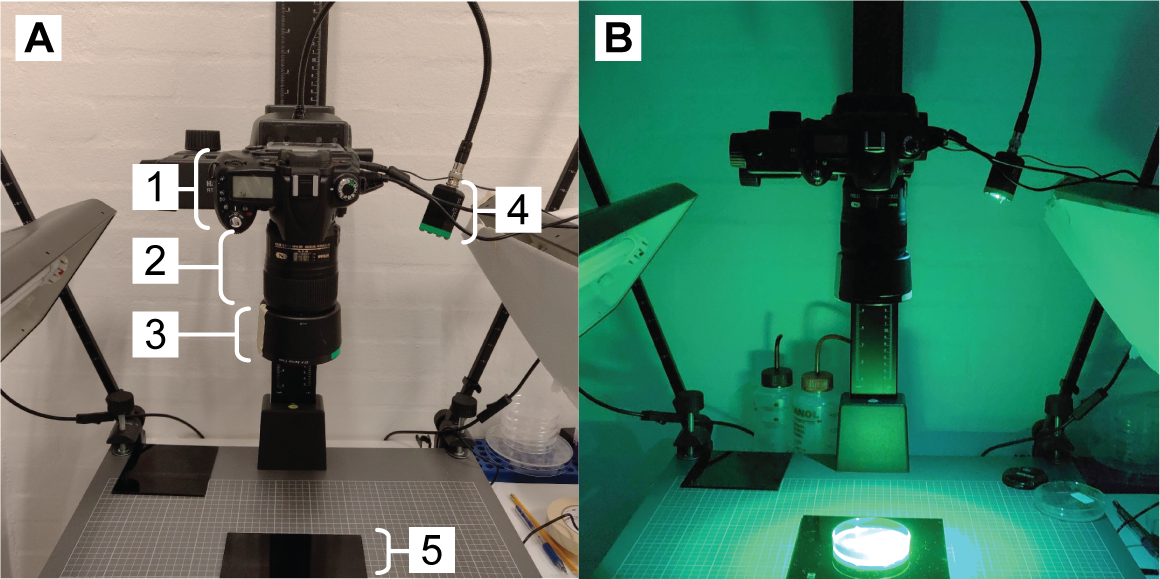


**Figure S6** Camera setup for qualitative plate screening by fluorescence photography. A) The components of the setup; 1 – Nikon D90 SLR camera, 2 – Nikon AF-S Micro NIKKOR 60mm lens, 3 – lens attachment equipped with red filter (Nightsea™ ), 4- green light source (Nightsea™), 5 – black glass background. B) The camera setup in operational mode.
