# Supplementary material for: Cpf1 enables fast and efficient genome editing in Aspergilli
Source: Fungal Biol Biotechnol. 2019 May 1;6:6. doi: 10.1186/s40694-019-0069-6 (PMC6492335; doi:10.1186/s40694-019-0069-6)
Supplement: Supplementary file 7 — Additional file 7: Fig. S7. Diagnostic PCR validation of mRFP gene insertions into different genetic loci in A. nidulans and in A. niger. a Schematic drawing of the experimental setup. Small arrows indicate primers positions and Roman numbers indicate resulting PCR fragments. The expected fragment lengths for four different loci are summarized in the table. b Diagnostic PCR reactions analyzed by gel electrophoresis. Samples from all individual experiments are loaded as indicated above individual lanes. [file 40694_2019_69_MOESM7_ESM.docx]

**Figure S7** Diagnostic PCR validation of *mRFP* gene insertions into different genetic loci in *A. nidulans* and in *A. niger*. A) Schematic drawing of the experimental setup. Small arrows indicate primers positions and Roman numbers indicate resulting PCR fragments. The expected fragment lengths for four different loci are summarized in the table. B) Diagnostic PCR reactions analyzed by gel electrophoresis. Samples from all individual experiments are loaded as indicated above individual lanes.
