# Supplementary material for: Cpf1 enables fast and efficient genome editing in Aspergilli
Source: Fungal Biol Biotechnol. 2019 May 1;6:6. doi: 10.1186/s40694-019-0069-6 (PMC6492335; doi:10.1186/s40694-019-0069-6)
Supplement: Supplementary file 8 — Additional file 8: Fig. S8. TAPE experiment. Tests to assess protospacer efficiency of gRNAs targeting A. nidulans Anid_IS1 and A. niger Anig_IS1. Transformation of A. nidulans NHEJ proficient NID5 and NHEJ deficient NID1, as well as of A. niger NHEJ proficient NIG81 and NHEJ deficient NIG96 with empty Cpf1-CRISPR vector (pAC1430) or Cpf1-CRISPR-tRNA vectors encoding gRNAs as indicated. Plasmids used for each transformation are indicated below plates for NHEJ proficient strains and above plates for NHEJ deficient strains. [file 40694_2019_69_MOESM8_ESM.docx]

**Figure S8** TAPE experiment. Tests to assess protospacer efficiency of gRNAs targeting *A. nidulans Anid_IS1* and *A. niger Anig_IS1*. Transformation of *A. nidulans* NHEJ proficient NID5 and NHEJ deficient NID1, as well as of *A. niger* NHEJ proficient NIG81 and NHEJ deficient NIG96 with empty Cpf1-CRISPR vector (pAC1430) or Cpf1-CRISPR-tRNA vectors encoding gRNAs as indicated. Plasmids used for each transformation are indicated below plates for NHEJ proficient strains and above plates for NHEJ deficient strains.
