# Supplementary material for: Cpf1 enables fast and efficient genome editing in Aspergilli
Source: Fungal Biol Biotechnol. 2019 May 1;6:6. doi: 10.1186/s40694-019-0069-6 (PMC6492335; doi:10.1186/s40694-019-0069-6)
Supplement: Supplementary file 9 — Additional file 9: Table S1. Primer used in this study. [file 40694_2019_69_MOESM9_ESM.docx]

**Table S1** Primer used in this study

| **Primer ID** | **Sequence^A^ 5’🡪3’** | **Description** |
| --- | --- | --- |
| **Primers for Cpf1-CRISPR vector** | | |
| P174 | GGGTTTAAU GCTGAGGGTTTAATTAAGACCTCAGC CGAGACAGCAGAATCACC | Anid_Ptef1 FW (re-introduces the PacI/Nt.BbvCI USER cassette) |
| P187 | ACGAAGTCU GTGTTATGTTTTGTGGAA | Anid_Ptef1 RV |
| P188 | AGACTTCGU ATGTCCAAGCTCGAGAAG | AnidCO_Lb_cpf1 FW |
| P189 | AGTAGACTU TCAGACCTTGCGCTTCTTC | AnidCO_Lb_cpf1 RV |
| P57 | AAGTCTACU GCGGACATTCGATTTATG | Anid_Ttef1 FW |
| P58 | GGTCTTAAU GTATTGGGATGAATTTTGTATGC | Anid_Ttef1 RV |
| **Primers for Cpf1-CRISPR-tRNA vectors expressing gRNA** | | |
| P1 | CGTGCGAUGATCACATAGATGCTCGG | U3p FW |
| P2 | ATCTACACU TAGTAGAAATTTGCATCATCCGTGAATCG | U3p RV with LbCpf1 repeat |
| P3 | AGTGTAGAU ATGCTTGCTACGGACTGGACATA GCATCATTGGTCTAGTGG | yA-gRNA1 |
| P5 | AGTGTAGAU ATTGGCGGCGCTGCGCAAAAGGC GCATCATTGGTCTAGTGG | yA-gRNA2 |
| P6 | AGTGTAGAU CAGCAATGCTTCCATGCAATTCG GCATCATTGGTCTAGTGG | albA-gRNA1 |
| P7 | AGTGTAGAU AAGTCAACCAAGCTCAAGGTTCC GCATCATTGGTCTAGTGG | albA-gRNA2 |
| P120 | AGTGTAGAU CCGTGTCTGGAACTGGTAAAATC GCATCATTGGTCTAGTGG | *Anig*_IS1 FW gRNA_1 |
| P122 | AGTGTAGAU ATTGACTGGAAGTCTCAGTTCCA GCATCATTGGTCTAGTGG | *Anig*_IS1 FW gRNA_2 |
| P123 | AGTGTAGAU ACGATAACATCCCAAACCAACCC  GCATCATTGGTCTAGTGG | *Anig*_IS1 FW gRNA_3 |
| P124 | AGTGTAGAU GCCCTCAACATCGGCACCGACAT GCATCATTGGTCTAGTGG | *Anig*_IS1 FW gRNA_4 |
| P125 | AGTGTAGAU AGGAAATCATCAGGTCGCT GCAAGCATCATTGGTCTAGTGG | *Anid_IS1* FW gRNA_1 |
| P126 | AGTGTAGAU GTGCAATTCACGCTCATCGGTGT GCATCATTGGTCTAGTGG | *Anid_IS1* FW gRNA_2 |
| P127 | AGTGTAGAU ATGATTGCTGGTCTGTGTAAGCG GCATCATTGGTCTAGTGG | *Anid_IS1* FW gRNA_3 |
| P128 | AGTGTAGAU GGCCCTTCCAAGCTGGTCAACGG GCATCATTGGTCTAGTGG | *Anid_IS1* FW gRNA_4 |
| P129 | AGTGTAGAU TTTCTTCTCCTTCTGACATCAAT GCATCATTGGTCTAGTGG | *Anid_IS1* FW gRNA_5 |
| P4 | CACGCGAUACCCTGAGAAGATAGATG | U3t RV |
| **Primers for PCR fragments containing *mRFP*** | | |
| P151 | ATATCGGTGTAGAAAGTAAGCGTGCGTGAAACAGCCACCAGATCATAACCAACAAGAGTA ATTCCCTTGTATCTCTACACACAGG | FW Anig_IS1UP-RFP cassette -IS1DW |
| P152 | TGAGTGATTGGATGGGAAGCTGTAGGAACTGGTAGATATGAATTTGAGTACTGGGTACAG TCTTAATGCGATGCTTCCATTGCC | RV Anig_IS1UP-RFP cassette -IS1DW |
| P153 | GGCCGGGTCATAGGAACGCTGGAGTGGTAGATGCGGGGTACGGTGGGAAGCGCCATCTGG ATTCCCTTGTATCTCTACACACAGG | FW Anid_IS1UP-RFP cassette -IS1DW |
| P154 | AAGCTGCTTTACGTCCTTTGAATCGGACTCGCCTTAATGTGCTCTTTAGTGATTGTAGCG TCTTAATGCGATGCTTCCATTGCC | RV Anid_IS1UP-RFP cassette -IS1DW |
| P159 | TCGAAGCTGGCCTGCGCCGTCTGCTCCAAGCGAAGAATAGTACCATTGTCCAGTCCTTTT ATTCCCTTGTATCTCTACACACAGG | FW albA-gRNA1 RFP cassette |
| P160 | ACGATGCTCGTGAAGCGTGGGAAGAGCTTCCGATGAGACGGCGGGAGCTTCGCGATCTCT TCTTAATGCGATGCTTCCATTGCC | RV albA-gRNA1 RFP cassette |
| P161 | GTGTCGAGCCCCCGGTGATCTACGTCGATCCCGAATTCAACGGTTGGGTCAGCCTTAATT ATTCCCTTGTATCTCTACACACAGG | FW yA-gRNA2 RFP cassette |
| P162 | TCAACAAACTGTCCGTCAACTTCATATACCCACATTGGGTGATTGTCGACAGAAAAAGTT TCTTAATGCGATGCTTCCATTGCC | RV yA-gRNA2 RFP cassette |
| **Primers for diagnostic PCR and sequencing** | | |
| P190 | TCACCGAAGAAAGCCTTG | Geneart AspCOLbCpf1 int seq RV |
| P191 | GATCTGCTCGATTCCGTG | Geneart AspCOLbCpf1 int seq FW |
| P192 | CGTCCATCCTGCTAACTC | Genesart AspCOLbCpf1 int seq FW_2 |
| P135 | CATGTGTATAAAGTGTGCGTCTCAT | FW Seq check albA-gRNA1 |
| P136 | GTGCAGCTCAGAACACCAGTG | RV Seq check albA-gRNA1 / RFP in *albA* locus check |
| P139 | GCTCAGTTGCTGACGAAGGA | FW Seq check albA-gRNA2 |
| P140 | CGATCTCAACCCACAGAGTCTT | RV Seq check albA-gRNA2 |
| P145 | CGTTCTGGTATCACTCGCATTAC | FW Seq check yA -gRNA1 |
| P146 | AGATGTGTTCCTTCGAGGACG | RV Seq check yA-gRNA1 |
| P147 | CGTCCTCGAAGGAACACATCT | FW Seq check yA-gRNA2 |
| P148 | CTGATTGACATACGAGAGGATGG | RV Seq check yA-gRNA2 / RFP in *yA* locus check |
| P193 | CGTCGTCCAAGTCGAGAAG | RFP in IS1 locus check |
| P194 | GAGGTGGCGGCTTCGGAG | RFP in *Anid_IS1* locus check |
| P195 | CTAAGCTATTCTTCTGCTTCGCC | RFP check |
| P196 | CGAGCTTTCCCACTTCATCG | RFP check |
| P197 | CCTATGAGTCGTTTACCCAG | RFP in pigment locus check |
| P198 | GACTATCATCCGACAGACAGAACC | RFP in *Anig_IS1* locus check |

^A^ Color code: Annealing sequence, protospacer, homologous recombination sequence, PacI/Nt.BbvCI cassette, LBCpf1 direct repeat, custom USER overhangs, thymine-> uracil substitution
