# Supplementary material for: Cpf1 enables fast and efficient genome editing in Aspergilli
Source: Fungal Biol Biotechnol. 2019 May 1;6:6. doi: 10.1186/s40694-019-0069-6 (PMC6492335; doi:10.1186/s40694-019-0069-6)
Supplement: Supplementary file 10 — Additional file 10: Table S2. Plasmids used in this study. [file 40694_2019_69_MOESM10_ESM.docx]

**Table S2** Plasmids used in this study

| **Plasmid ID** | **Description** | **Reference** |
| --- | --- | --- |
| pAC572 | *Af_pyrG*-AMA1 PacI/Nt.BbvCI |  |
| pAC573 | *An_argB*-AMA1 PacI/Nt.BbvCI |  |
| pAC574 | *Ec_hygB*-AMA1 PacI/Nt.BbvCI |  |
| pAC575 | *Sh_ble*-AMA1 PacI/Nt.BbvCI |  |
| pAC902 | *Af_pyrG*-AMA1 *yA-sgRNA1 Sp_cas9* | [17] |
| pAC1430 | *Af_pyrG*-AMA1_PacI/Nt.BbvCI *Lb_cpf1* | This study |
| pAC1748 | *An_argB* -AMA1_PacI/Nt.BbvCI *Lb_cpf1* | This study |
| pAC1749 | *Ec_hygB* -AMA1_PacI/Nt.BbvCI *Lb_cpf1* | This study |
| pAC1750 | *Sh_ble* -AMA1_PacI/Nt.BbvCI *Lb_cpf1* | This study |
| pAC1441 | *Af_pyrG*-AMA1 *yA-gRNA1* *Lb_cpf1* | This study |
| pAC1442 | *Af_pyrG*-AMA1 *yA-gRNA2* *Lb_cpf1* | This study |
| pAC1443 | *Af_pyrG*-AMA1 *albA-gRNA1 Lb_cpf1* | This study |
| pAC1444 | *Af_pyrG*-AMA1 *albA-gRNA2 Lb_cpf1* | This study |
| pAC1445 | *Af_pyrG*-AMA1 *Anig_IS1-gRNA1 Lb_cpf1* | This study |
| pAC1446 | *Af_pyrG*-AMA1 *Anig_IS1-gRNA2 Lb_cpf1* | This study |
| pAC1447 | *Af_pyrG*-AMA1 *Anig_IS1-gRNA3 Lb_cpf1* | This study |
| pAC1448 | *Af_pyrG*-AMA1 *Anig_IS1-gRNA4 Lb_cpf1* | This study |
| pAC1449 | *Af_pyrG*-AMA1 *Anid_IS1-gRNA1 Lb_cpf1* | This study |
| pAC1450 | *Af_pyrG*-AMA1 *Anid_IS1-gRNA2 Lb_cpf1* | This study |
| pAC1451 | *Af_pyrG*-AMA1 *Anid_IS1-gRNA3 Lb_cpf1* | This study |
| pAC1452 | *Af_pyrG*-AMA1 *Anid_IS1-gRNA4 Lb_cpf1* | This study |
| pAC1453 | *Af_pyrG*-AMA1 *Anid_IS1-gRNA5 Lb_cpf1* | This study |
