# Supplementary material for: Cpf1 enables fast and efficient genome editing in Aspergilli
Source: Fungal Biol Biotechnol. 2019 May 1;6:6. doi: 10.1186/s40694-019-0069-6 (PMC6492335; doi:10.1186/s40694-019-0069-6)
Supplement: Supplementary file 11 — Additional file 11: Fig. S9. Sequence of A. nidulans codon optimized Lb_cpf1. [file 40694_2019_69_MOESM11_ESM.docx]

***A .nidulans* codon optimized Lb_cpf1**

**ATGAGCAAGCTTGAGAAGTTCACCAACTGTTATTCCCTTTCGAAGACACTTAGATTCAAAGCCATTCCAGTGGGCAAAACGCAGGAAAATATTGATAACAAGAGACTTCTCGTTGAAGACGAGAAGAGGGCGGAAGACTACAAGGGCGTAAAGAAGTTGCTCGATCGGTATTATCTTTCTTTCATCAACGATGTGCTGCACTCAATAAAATTGAAGAACTTGAATAATTACATAAGTTTGTTCAGGAAAAAAACGCGAACAGAAAAGGAAAACAAGGAACTCGAAAATCTTGAAATTAACTTGCGAAAGGAAATCGCAAAAGCCTTCAAGGGGAATGAAGGTTATAAGAGCTTGTTCAAAAAAGATATTATCGAGACCATTCTCCCAGAATTTTTGGATGACAAAGACGAAATCGCACTCGTAAATTCTTTTAATGGGTTTACAACCGCTTTTACTGGTTTTTTCGACAATCGCGAAAACATGTTTTCCGAAGAGGCCAAGTCCACTTCCATTGCGTTCCGTTGTATCAATGAAAATCTGACAAGGTATATTAGCAACATGGATATTTTCGAGAAAGTAGATGCGATCTTTGACAAGCATGAAGTTCAGGAGATAAAAGAGAAAATCCTGAATTCCGATTACGACGTGGAGGACTTCTTCGAAGGGGAATTCTTCAACTTCGTTTTGACGCAAGAAGGTATCGACGTCTATAATGCAATTATCGGAGGTTTCGTGACGGAGTCGGGGGAGAAGATAAAGGGTCTTAATGAGTACATCAATTTGTATAACCAGAAAACCAAACAGAAGCTGCCAAAGTTCAAGCCACTCTATAAGCAAGTCCTTTCGGACCGGGAATCATTGTCCTTCTACGGCGAGGGATATACGTCTGATGAAGAAGTGCTGGAAGTCTTCCGAAATACACTGAACAAAAACTCAGAAATCTTTTCCAGTATCAAAAAACTGGAAAAGCTGTTCAAGAATTTCGATGAGTATTCATCAGCCGGAATCTTCGTGAAAAACGGCCCTGCAATTAGTACTATATCTAAAGACATTTTCGGTGAATGGAATGTTATAAGGGATAAATGGAACGCCGAATACGATGACATTCATTTGAAGAAAAAGGCGGTCGTAACAGAGAAGTATGAGGATGATAGAAGGAAGTCCTTCAAAAAGATTGGTAGCTTCTCACTCGAACAATTGCAGGAGTACGCCGATGCCGACCTTAGTGTAGTCGAAAAGCTTAAAGAGATCATAATCCAAAAGGTCGACGAAATTTATAAGGTCTACGGCAGTAGCGAGAAACTTTTCGATGCCGATTTTGTGTTGGAGAAGAGTCTGAAGAAAAATGACGCAGTGGTGGCGATTATGAAAGACTTGCTGGATAGCGTTAAAAGTTTTGAAAATTATATCAAGGCGTTTTTTGGAGAAGGGAAGGAAACCAATCGTGACGAGTCTTTTTATGGCGATTTTGTATTGGCATATGATATCCTTCTGAAGGTCGATCATATATACGACGCAATACGAAACTACGTGACCCAAAAGCCGTATTCGAAGGATAAGTTCAAACTGTACTTTCAGAACCCACAGTTCATGGGAGGCTGGGACAAGGACAAGGAAACAGACTACAGGGCCACAATTCTTCGGTACGGATCTAAGTATTATCTTGCGATCATGGACAAGAAGTATGCCAAATGCTTGCAAAAGATAGACAAAGATGACGTAAATGGGAATTACGAAAAGATTAATTATAAACTCCTGCCAGGACCGAATAAAATGTTGCCCAAGGTATTTTTCAGTAAAAAATGGATGGCATATTACAACCCAAGCGAGGACATCCAGAAAATATATAAGAACGGGACGTTTAAGAAGGGGGACATGTTTAATTTGAATGACTGTCACAAATTGATAGACTTTTTCAAAGATAGTATCTCGAGGTATCCAAAATGGTCAAATGCTTACGATTTCAACTTCTCGGAAACGGAGAAATACAAAGATATCGCAGGATTCTATAGGGAAGTGGAAGAACAAGGCTATAAGGTGAGTTTTGAGAGTGCCTCTAAGAAGGAGGTGGACAAACTCGTGGAAGAAGGAAAATTGTATATGTTCCAAATATATAACAAGGACTTCTCTGACAAGTCTCATGGGACTCCTAACTTGCATACGATGTACTTTAAACTCCTGTTTGATGAGAACAACCACGGTCAGATTCGTCTGAGCGGGGGCGCGGAACTTTTTATGCGTCGTGCAAGCCTGAAGAAGGAAGAACTGGTCGTACATCCGGCAAACTCTCCTATCGCTAACAAGAACCCTGATAATCCTAAAAAGACCACAACTCTCTCTTATGATGTGTATAAAGATAAGCGGTTTAGTGAGGATCAGTACGAGCTTCACATTCCGATCGCTATTAACAAGTGCCCTAAAAATATTTTTAAAATAAATACAGAGGTTCGCGTGTTGCTCAAGCATGACGATAACCCGTATGTAATCGGAATTGACAGAGGGGAGCGCAATCTCCTTTACATCGTTGTAGTAGATGGAAAAGGAAACATAGTAGAGCAATATTCCTTGAATGAGATCATTAATAACTTCAACGGCATCCGCATAAAAACCGACTATCATAGCCTTCTGGACAAAAAGGAGAAAGAACGTTTTGAAGCAAGGCAGAATTGGACTTCGATCGAGAATATCAAGGAATTGAAGGCAGGATACATCAGTCAGGTGGTACATAAAATTTGCGAGTTGGTTGAAAAATATGATGCAGTCATAGCCCTCGAAGATTTGAACTCTGGGTTTAAGAATTCCAGGGTCAAAGTCGAAAAACAGGTCTATCAGAAATTCGAGAAAATGCTCATTGACAAGCTCAATTATATGGTAGACAAGAAGTCCAACCCGTGTGCTACTGGGGGAGCACTTAAAGGGTATCAAATAACCAACAAGTTTGAAAGTTTTAAGTCTATGTCAACGCAGAACGGATTTATTTTCTACATACCAGCGTGGCTGACATCGAAGATCGATCCATCAACGGGATTCGTTAACTTGCTTAAAACAAAGTACACTTCGATTGCAGACTCTAAAAAGTTTATATCTTCGTTCGATAGGATAATGTACGTACCGGAGGAAGATCTCTTCGAGTTTGCTCTCGACTATAAAAACTTTTCGCGAACGGATGCGGACTACATAAAAAAGTGGAAACTTTACAGCTATGGGAACCGAATAAGGATATTCCGAAACCCAAAAAAGAACAACGTCTTTGATTGGGAAGAGGTCTGTTTGACCTCTGCCTATAAGGAACTGTTCAACAAGTACGGGATCAACTATCAACAAGGTGATATAAGGGCGTTGCTTTGTGAACAGTCAGACAAAGCGTTTTATTCATCTTTCATGGCCCTCATGTCCTTGATGCTGCAAATGAGGAATTCGATAACGGGCAGGACGGACGTCGACTTCCTTATTAGCCCTGTGAAAAACAGTGACGGAATTTTCTATGACAGCCGTAATTACGAAGCTCAGGAGAACGCAATCCTTCCAAAAAATGCGGATGCCAATGGAGCTTATAATATTGCTAGAAAGGTTCTGTGGGCCATTGGACAGTTTAAAAAGGCTGAAGATGAAAAACTGGACAAGGTTAAAATCGCTATATCTAACAAGGAGTGGCTGGAATACGCGCAAACGAGTGTAAAACATAAACGGCCGGCCGCCACGAAAAAAGCTGGGCAAGCTAAAAAGAAAAAGGGTAGCTACCCATATGATGTGCCAGATTATGCGTACCCATACGACGTTCCGGATTACGCTTACCCTTATGATGTACCTGACTACGCTCCCCCCAAGAAGAAGCGCAAGGTCTGA**

**NNN –** SV40 Nuclear Localization Signal

**NNN –** Stop codon (opal)

**Figure S9** Sequence of *A. nidulans* codon optimized *Lb_cpf1*.
