# Supplementary material for: Cpf1 enables fast and efficient genome editing in Aspergilli
Source: Fungal Biol Biotechnol. 2019 May 1;6:6. doi: 10.1186/s40694-019-0069-6 (PMC6492335; doi:10.1186/s40694-019-0069-6)
Supplement: Supplementary file 12 — Additional file 12: Table S3. Oligonucleotides used in this study. [file 40694_2019_69_MOESM12_ESM.docx]

**Table S3** Oligonucleotides used in this study

| **Oligo ID** | **Sequence** | **Description** |
| --- | --- | --- |
| *oKST_155* | CGTCTGCTCCAAGCGAAGAATAGTACCATTGTCCAGTCCTtctagaGAGATCGCGAAGCTCCCGCCGTCTCATCGGAAGCTCTTCCCACG | albA-gRNA1 XbaI |
| *oKST_157* | GAGAGAGTTAGCAGAAATACAGTACGCAGAAGATAATCCTTATtctagaCTTCGGCGGAGTATCATAACATCGAGGTTGAGTCTGGCTAT | yA-gRNA1 XbaI |
| *oKST_158* | ATCTACGTCGATCCCGAATTCAACGGTTGGGTCAGCCTTAATtctagaTAACTTTTTCTGTCGACAATCACCCAATGTGGGTATATGAAG | yA-gRNA2 XbaI |
